# Inhibitory Effect of Piceatannol on *Streptococcus suis* Infection Both *in vitro* and *in vivo*

**DOI:** 10.3389/fmicb.2020.593588

**Published:** 2020-11-27

**Authors:** Guizhen Wang, Yawen Gao, Xiuhua Wu, Xiue Gao, Min Zhang, Hongmei Liu, Tianqi Fang

**Affiliations:** ^1^Department of Respiratory Medicine, The First Hospital of Jilin University, Jilin University, Changchun, China; ^2^College of Food Engineering, Jilin Engineering Normal University, Changchun, China; ^3^Key Laboratory of Zoonosis Research, Ministry of Education, College of Veterinary Medicine, Jilin University, Changchun, China

**Keywords:** *Streptococcus suis*, piceatannol, suilysin, virulence, molecular modeling

## Abstract

Suilysin (SLY) plays a critical role in *Streptococcus suis* infections making it an ideal target to the combat infection caused by this pathogen. In the present study, we found that piceatannol (PN), a natural compound, inhibits pore-formation by blocking the oligomerization of SLY without affecting the growth of *S. suis* and the expression of SLY. Furthermore, PN alleviated the J774 cell damage and the expression of the inflammatory cytokine tumor necrosis factor-α (TNF-α) and interleukin-1α (IL-1β) induced by *S. suis in vitro*. The computational biology and biochemistry results indicated that PN binds to the joint region of D2 and D4 in SLY, and Asn57, Pro58, Pro59, Glu76, Ile379, Glu380, and Glu418 were critical residues involved in the binding. The binding effect between PN and SLY hindered the SLY monomers from forming the oligomers, thereby weakening the hemolytic activity of SLY. This mechanism was also verified by hemolysis analysis and analysis of *K_A_* formation after site-specific mutagenesis. Furthermore, PN protected mice from *S. suis* infections by reducing bacterial colony formation and the inflammatory response in target organs *in vivo*. These results indicate that PN is a feasible drug candidate to combat *S. suis* infections.

## Introduction

*Streptococcus suis* is a common Gram-positive bacterium that can cause a variety of infectious diseases in humans and pigs, including meningitis, arthritis, septicaemia, and pneumonia (Ajaree et al., 2018; Agoston et al., 2020). *Streptococcus suis* infection causes huge economic losses to the pig industry every year and seriously affects the health and development of the pig industry (Haas and Grenier, 2018). In addition, swine are important food-producing animals; *S. suis* induces human infection through infected pigs or their products. *Streptococcus suis* infection in humans is distributed globally and poses a serious threat to public health (Huong et al., 2014; Agoston et al., 2020; Olearo et al., 2020). With the development of bacterial resistance, great challenges in fighting with infections induced by *S. suis* have been encountered by humans (Seitz et al., 2016; Ma et al., 2018; Che et al., 2019). Therefore, the development of new drugs against this pathogen is of great significance for its prevention and control.

With the development of molecular biology, the role of virulence factors in bacterial infection has been gradually discovered and clarified. It is highly apparent that bacterial pathogenicity can be reduced by interfering with bacterial virulence factors (Maura et al., 2016; Mühlen and Dersch, 2016; Theuretzbacher and Piddock, 2019). Suilysin (SLY) is a critical toxin of *S. suis* and has been demonstrated to promote *S. suis* infection through a variety of aspects (Tenenbaum et al., 2016). The basic biological function of SLY is the generation of pores on cell membranes by forming oligomers, causing cytotoxicity (Lun et al., 2003). In addition, SLY has been demonstrated to be related to the inflammatory response induced by *S. suis* through promoting the release of inflammatory factors such as TNF-α and IL-1β, which involve in TLR4 and p38-MAPK signaling pathway (Vadeboncoeur et al., 2003; Bi et al., 2015). *Streptococcus suis* that produce SLY could cause severe damage to epithelial cells, and the injury manifests as the loss of the cytoplasmic density, discontinuity of the cytoplasmic membranes, and disappearance of the nucleus (Lalonde et al., 2000). The cytotoxic effects of SLY on porcine and human brain microvascular endothelial cells have been confirmed, and SLY is involved in the adhesion and invasion of *S. suis* in human respiratory epithelial cells under certain conditions; SLY-positive strains could invade the epithelial cells of the host at a higher percentage than SLY-negative strains (Norton et al., 2010; Seitz et al., 2013). In an intraperitoneal mouse infection model, SLY was shown to be critical for the development of bacterial meningitis and bacterial survival (Takeuchi et al., 2014). Therefore, SLY has an essential effect on the pathogenesis of *S. suis*.

The SLY protein exists as soluble monomers and binds to cholesterol on the membranes of host cells, thereby assembling large, oligomeric pores on membranes (Tweten, 2005). The research group of Xu et al. (2010) analyzed the crystal structure of the SLY monomers based on X-ray crystallography. The monomeric SLY protein contains 497 amino acids and has an α-carbon skeleton structure composed of four discontinuous domains (D1–D4); D1–D3 are discontinuous in the primary sequence, but D4, in contrast, is a continuous domain formed by the C-terminus of SLY (Xu et al., 2010). The bioactive function of SLY is closely related to its spatial structure, which changes directly to result in the reduction or loss of its biological function. D4 promotes the initial binding of SLY to the cholesterol-containing membrane (Weis and Palmer, 2001; Shimada et al., 2002); it recognizes cholesterol on the host cell membranes and binds specifically to its receptor (Farrand et al., 2010). This process is mediated *via* the C-terminal tryptophan-rich undecapeptide of D4 (Rossjohn et al., 1998), which comprises of 11 amino acids (ECTGLAWEWWR). SLY monomers diffuse in all directions to form a pre-pore complex (Shepard et al., 2000), and the pre-pore complexes form pore complexes (known as oligomer) *via* a series of conformational changes (Hotze et al., 2012). Subsequently, the two β-hairpins of D3 in each SLY monomer are assembled cooperatively and form an oligomer β-barrel pore that can penetrate the host cell membrane (Shepard and Heuck, 1998; Shatursky, 1999). In the final step, D2 acts as a “hinge joint,” promoting close contact between D3 and the host cell surface. The β-barrel structure that forms during oligomerization inserts itself into the host cell membrane and generates a transmembrane pore along with the “collapse” of D2. The oligomer generated in the process is usually comprised of 30–50 monomers and forms an orifice with a diameter of 250–350 Å (Gilbert, 2005; Tweten, 2005), which is permeable for ions and macromolecules (Leung et al., 2014).

The crucial role of SLY in the pathogenicity of *S. suis* makes it an ideal potential target for identifying inhibitors to combat infectious diseases caused by this pathogen. Therefore, the discovery of novel SLY inhibitors and the confirmation of their interaction mechanism may provide useful information for the therapy of *S. suis* infections. Piceatannol (PN), a natural compound that exists in various fruits and herbs, such as grapes blueberries and giant knotweed, etc. (Piotrowska et al., 2012; Wen et al., 2018), has been demonstrated possessing multiple health-enhancing properties, including anti-cancer, anti-oxidant, and anti-inflammatory activities (Piotrowska et al., 2012; Jin et al., 2018). However, the effect of PN against *S. suis* infection has not been reported. In the present study, we found that the natural compound PN could bind with SLY directly, causing spatial conformation changes and blocking the generation of SLY oligomers, finally resulting in the loss of the hemolytic activity of SLY. Furthermore, the cytotoxicity and the inflammatory response were significantly alleviated both *in vitro* and *in vivo* after infected cells or mice received PN treatment, but PN did not exhibit antibacterial properties. The expression of SLY was unaffected. These results indicate that PN could be a potential lead compound used to develop new pharmaceuticals for fighting *S. suis* infections.

## Materials and Methods

**Figure 1:**
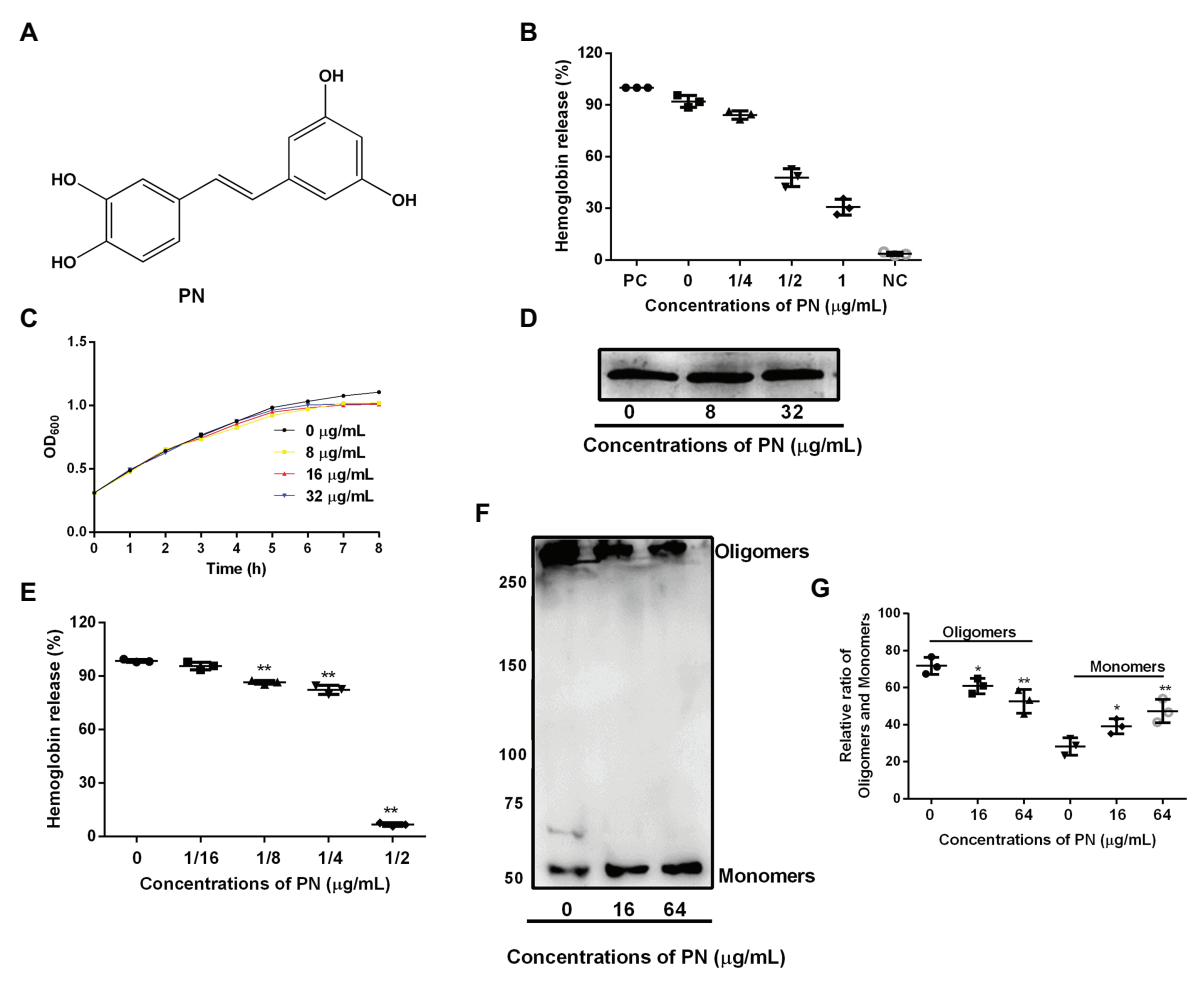
The inhibitory effects of piceatannol (PN) on suilysin (SLY) or the culture supernatant of *Streptococcus suis*. **(A)** The chemical structure of PN. **(B)** The inhibitory effect of PN on bacterial culture supernatants after coculture with SS2. PC represents positive control, NC represents negative control. **(C)** The growth of SS2 in the presence of the indicated PN concentrations. **(D)** The SLY expression level. **(E)** The inhibitory effect of PN on SLY-induced hemolysis. **(F)** The formation of SLY oligomers after treatment with different concentrations of PN. **(G)** The relative ratio of oligomers and monomers of the total protein in the oligomerization assays. All data are shown as the mean ± SD (*n* = 3), ^*^indicates *p* < 0.05, ^**^indicates *p* < 0.01. An unpaired two-tailed *t*-test was used for statistical analysis of the data.

Piceatannol (Figure 1A) was obtained from Chengdu LookChem Co., Ltd. (Chengdu, China). The highly virulent serotype 2 of *S. suis* (Number ZY05719) was obtained from Nanjing Agricultural University. The initial X-ray crystal structure of SLY (PDB number 3HVN) used for mechanistic research was from the Protein Data Bank (PDB). ChemDraw was used to draw the PN structure. The structure of the SLY-PN complex used for subsequent molecular dynamics (MD) simulations was obtained from the molecular docking result.

### Hemolysis Assays

*Streptococcus suis* that cultured overnight was transferred (1:100) to Brain-Heart Infusion broth with Yeast (BHY) extract added with various concentrations of PN (0, 0.25, 0.5, or 1 μg/ml) and further cultured until it reached the stationary phase. Then, the bacteria were pelleted by centrifugation to obtain the culture supernatants. Defibrinated sheep blood (2.5% vol) was incubated for 30 min with the culture supernatant at 37°C. Then, each sample was used to detect the absorbency of 543 nm (OD_543_) after centrifuge (13,800 *g* for 2 min) by using a microplate reader. Blood cell in PBS or 1% Triton X-100 was set as negative control (NC) or positive control (PC). The hemoglobin release was calculated based on the equation:

$$(\text{O}{\text{D}}_{543\text{sample}}-\text{O}{\text{D}}_{543\text{negative}})/(\text{O}{\text{D}}_{543\text{positive}}-\text{O}{\text{D}}_{543\text{negative}})\times 100.$$

For SLY protein (obtained from our laboratory; Li et al., 2017), the protein was incubated with different concentrations of PN (0, 0.0625, 0.125, 0.25, or 0.5 μg/ml) at 37°C for 20 min, and then defibrinated sheep blood was supplement to reach a concentration of 2.5% and cocultured for 10 min at the same temperature. Equal amounts of supernatants were obtained to detect the OD_543_ after centrifugation.

### Minimal Inhibitory Concentration

SS2 (final concentration 5 × 10^5^ CFUs/ml) was cocultured with different concentrations of PN (1–1,024 μg/ml) in BHY media at 37°C for 24 h. The concentration at which no visible bacterial growth was observed was defined as the minimal inhibitory concentration (MIC) of PN.

### Growth Curves

SS2 was cultured overnight and was diluted (1:100), and it was further cultured until the OD_600_ reached 0.3, after which it was sub-packaged and cocultured with the expected concentrations of PN (0, 8, 16, or 32 μg/ml). The OD_600_ was detected every hour until the stationary phase was reached.

### Detection of the Expression Level of SLY

After culturing overnight, SS2 was diluted and cocultured with different concentrations of PN (0, 8, or 32 μg/ml) until the stationary phase was reached. After centrifugation (1,500 *g*, 5 min), equal amounts of supernatants were added to sodium dodecyl sulfate-polyacrylamide gel electrophoresis (SDS-PAGE) loading buffer and treated at 100°C for 10 min. Then, an equal volume of sample was applied to the protein electrophoresis gel. After separation, the protein was transferred to polyvinylidene difluoride (PVDF) membranes (Bio-Rad, Hercules, CA, United States). After blocking with 5% skimmed milk powder for 2 h, the membranes were incubated with SLY antibody (1:800) that obtained from Tianjin Sungene Biotech Co., Ltd. (Tianjin, China) for 2 h. Subsequently, the membranes were incubated with the secondary antibody (1:4,000, obtained from Sigma-Aldrich), which was linked to horseradish peroxidase after washing with phosphate buffer containing Tween 20 (PBST). Finally, the membrane containing the protein of interest was treated with ECL (GE Healthcare, Buckinghamshire, United Kingdom) luminescent solution and observed with a gel imaging system.

### The Inhibition of Oligomer Formation

The purified SLY protein (obtained from our laboratory) was incubated with different concentrations of PN (0, 16, or 64 μg/ml) for 20 min at 37°C. Potassium chloride was added to induce oligomerization *in vitro*, and then the loading buffer without β-mercaptoethanol was added, and treated for 10 min at 55°C. Subsequently, an equal volume of sample was added to 6% SDS-PAGE. The rest of the steps are the same as those used for the expression of SLY.

### Cytotoxicity Assays

Mouse J774 cells (serial number: BFN60807356, stored in our laboratory, obtained from ATCC) was cultured in Dulbecco’s modified eagle medium (DMEM) with 10% fetal bovine serum (FBS) and 100 IU/ml of penicillin and streptomycin. Cells were seeded in 96-well plate (5 × 10^4^ cells/well) and cultured overnight. The second day, the cells were treated with SLY protein (0.8 μg/ml) or SS2 (MOI = 20) resuspended in FBS-free DMEM medium, and received various concentrations of PN (0, 8, or 32 μg/ml) treatment. Five hours later, equal volume sample was used to interact with a cytotoxicity detection kit (Roche, Basel, Switzerland) for 30 min after centrifuge (100 *g* for 10 min). OD_490_ of each sample was used to analyze the lactate dehydrogenase (LDH) release in the system. PC or NC was set as cells received 2.5% Triton X-100 or DMEM, respectively.

### Detection of Inflammatory Factors

Mouse macrophage J774 cells infected with SS2 (the MOI was 5) were treated with or without PN (32 μg/ml) and coincubated for 4 h. Then, the culture medium was harvested by centrifugation (4°C, 100 *g*, 5 min). The cytokines (TNF-α and IL-1β) were detected using an enzyme-linked immunosorbent assay (ELISA) kit (Invitrogen, Thermo Fisher). PC or NC was set as cells received by *S. suis* or DMEM, respectively.

### QM Calculation

The ground state geometrical structure of PN was optimized using Gaussian 09 software with the DFT method (B3LYP exchange correlation functional approach) and the 6-31G (d, p) basis set for C, N, O, and H atoms. The geometry of the inhibitor was fully optimized in the gas phase without any symmetry constraints. Then, the frequency calculation was performed to check whether the computed vibrational frequencies were positive numbers and that no imaginary frequencies existed, confirming that the optimized geometry of the inhibitor represented the local minimum.

### Molecular Dynamics Simulation

Before docking, we performed a 100-ns MD simulation of SLY to obtain a more stable SLY crystal structure, which was used to obtain the initial coordinates for the molecular docking calculations using AutoDock 4.0 (Hu et al., 2010).

The simulation and analyses of the trajectories were performed with Gromacs 5.0.1 software (Hess et al., 2008) using the Amber99SB force field and the TIP3P water model (Jorgensen et al., 1983). The LINCS (Ryckaert et al., 1977) algorithm was used to constrain all of the bond lengths. For the water molecules, the SETTLE algorithm (Ryckaert et al., 1977) was used. The parameters of PN were estimated with the antechamber package (Wang et al., 2006) and the RESP partial atomic charges from the Amber suite (Jakalian et al., 2002). Analysis of the trajectories was performed by using the VMD, PyMOL, and Gromacs analysis tools.

The binding free energies were calculated using the Molecular Mechanics/Poisson-Boltzman Surface Area (MM-PBSA) approach (Punkvang et al., 2010; Schaffner-Barbero et al., 2010) with the Amber 10 package. Other details were according to the methods described previously (Dong et al., 2013; Qiu et al., 2013; Gao et al., 2020).

### Site-Specific Mutagenesis Assays

We carried out PCR assays by using plasmid carrying SLY protein fragment as a template to obtain site-directed mutant plasmids of P58A and E76A. These mutant plasmids were transferred to DH5α for cloning after demethylation at 37°C for 2 h. *Escherichia coli* BL21 (DE3) was used to express these mutant proteins. The purification of the proteins was based on the method described previously (Li et al., 2017). The primers used in this assay are shown in Table 1. The inhibitory effects of PN on the pore-forming activities of the mutant proteins were evaluated by using the method described for the hemolytic activity assay above. Hemocyte treated with proteins or PBS buffer was set as PC or NC.

**Table 1:** Primers used in site-specific mutagenesis study.

Name	Oligonucleotide (5'-3')^*^
SLY-E76A-F	GTACTTCGCAGAGCGAAGAAGAATATTAC
SLY-E76A-R	GTAATATTCTTCTTCGCTCTGCGAAGTAC
SLY-P58A-F	GAATACATTGATAATGCGCCAGCAACAACTG
SLY-P58A-R	CAGTTGTTGCTGGCGCATTATCAATGTATTC

* The underlined basic group represents mutated codons.

### Animal Assays

Female BALB/c mice that were 6–8-week-old were obtained from Liaoning Changsheng biotechnology Co.Ltd. (Liaoning, China), and all the procedures used in this assay were performed according to the guidelines established by the Animal Care and Use Committee (ACUC) of Jilin University. *Streptococcus suis* in mid-log phase was used to infect mice through intraperitoneal injection (1 × 10^8^ CFU/mouse). Three groups were utilized. Two hours later, infected mice that received the PN (80 mg/ml dissolved in DMSO, 25 μl/mice) cure through subcutaneous injection (100 mg/kg) were defined as the cure group, and the infected or healthy mice that received an equal volume of DMSO was used as the positive or blank control group. Two days later, the mice were euthanized with anesthesia, and the liver and spleen tissues harvested from the tested mice were subjected to homogenization processing in sterile PBS and colony counting after inoculation onto BHY agar plates to evaluate the bacterial burden in these tissues. The tissue homogenate supernatant was used to perform ELISA assays after centrifugation.

### Statistical Analysis

The experimental data are presented as the mean ± SD. Three independent experiments were performed. SPSS 17.0 was used for the statistical analysis by using the unpaired two-tailed *t*-test, and *p* < 0.05 was considered as statistically significant.

## Results

### The Inhibitory Effects of PN on SLY or the Culture Supernatant of *S. suis*

Around 92.06% hemoglobin was released when blood cells were treated with *S. suis* culture supernatant without PN (Figure 1A) treatment compared with positive group, indicating that the blood cells were lysed. In contrast, the hemoglobin release decreased when the supernatant of *S. suis* that was cultured with various concentrations of PN (Figure 1B), indicating that PN inhibited the hemolytic ability of the culture supernatant of *S. suis* in a dose-dependent manner. Furthermore, we confirmed that PN did not affect the growth of *S. suis* (Figure 1C; MIC value 256 μg/ml) or the expression levels of SLY (Figure 1D). These results indicated that the inhibition by PN to the hemolytic abilities of *S. suis* culture supernatant was not achieved by inhibiting the growth of *S. suis* or the expression of SLY. SLY shows pore-forming activity by forming oligomers (Tenenbaum et al., 2016). Here, we found that PN inhibited the hemolytic ability of purified SLY protein (Figure 1E) and the formation of oligomers reduced after SLY was coincubated with different concentration PN (Figures 1F,G). Taken together, these results suggested that PN inhibited the pore-forming activity of SLY by affecting the formation of its oligomers but did not affect *S. suis* growth or the expression of SLY.

### PN Alleviates *S. suis*-Mediated Cell Injury and Inflammation by Targeting SLY

**Figure 2:**
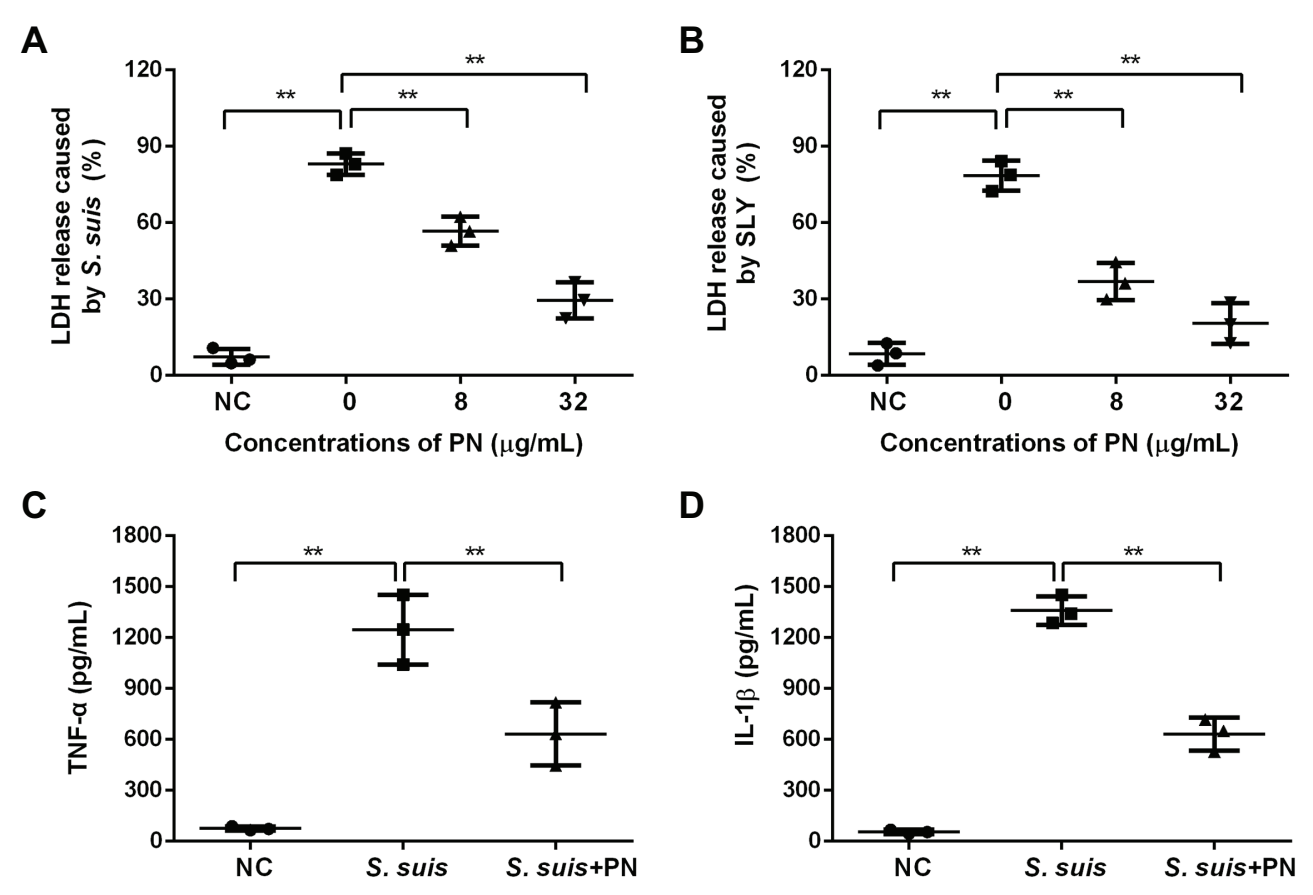
PN protected cells from injury and inflammation induced by *S. suis*. **(A)** The lactate dehydrogenase (LDH) release from cells treated with *S. suis* or SLY. **(B)** along with different concentrations of PN. **(C)** The levels of TNF-α and IL-1β **(D)** in the infected cells treated with or without PN. All data are shown as the mean ± SD (*n* = 3), ^**^ indicates *p* < 0.01. An unpaired two-tailed *t*- test was used for statistical analysis of the data.

To evaluate whether PN could reduce the cytotoxicity and inflammation mediated by *S. suis*, the protective effect of PN on infected cells was analyzed by LDH release and ELISA assays. With the increase in the PN concentration in the coinfection system, the activity of LDH decreased, indicating that PN can significantly reduce the damage to cells caused by SLY and *S. suis* (Figures 2A,B). Subsequently, the levels of TNF-α and IL-1β in the coinfection system were used to explore the effect of PN on alleviating *S. suis*-induced inflammation. After infection of J774 cells with *S. suis*, a large number of TNF-α and IL-1β were produced, indicating that *S. suis* triggered a severe inflammatory response in cells. However, when the infected cells were treated with PN, the levels of inflammatory factors in the coinfection system were significantly decreased (Figures 2C,D). In short, PN treatment alleviated the damage and inflammatory response caused by *S. suis*.

### Determination of the Binding Mode of PN and SLY

**Figure 3:**
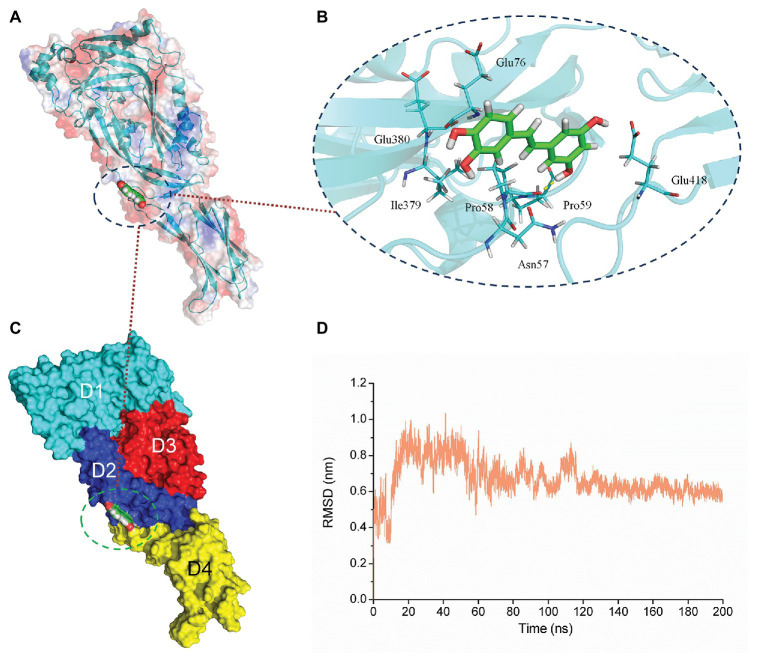
The equilibrium of the PN-SLY complex system. **(A,C)** The binding mode of SLY with PN from molecular dynamics (MD) simulation **(B)** The details of residues that play roles during the binding process. **(D)** The root-mean-square-deviation (RMSD) values of the SLY-PN complex during MD simulation.

According to the results of the SLY activity assay, we hypothesized that PN might directly interact with SLY. Therefore, we performed molecular docking and molecular modeling assays to explore the exact mechanism. After a 200-ns simulation, the 3D structure of the SLY-PN complex was determined (Figure 3). The equilibrium of the complex system was verified based on the root-mean-square-deviations (RMSDs) of the SLY-PN complex. As shown in Figure 3D, the RMSD value was between 0.5 and 0.7 nm after 120 ns, indicating that the complex system reached equilibrium at 120 ns. Thus, the simulation after 80 ns could be reliably used for the following analysis.

**Figure 4:**
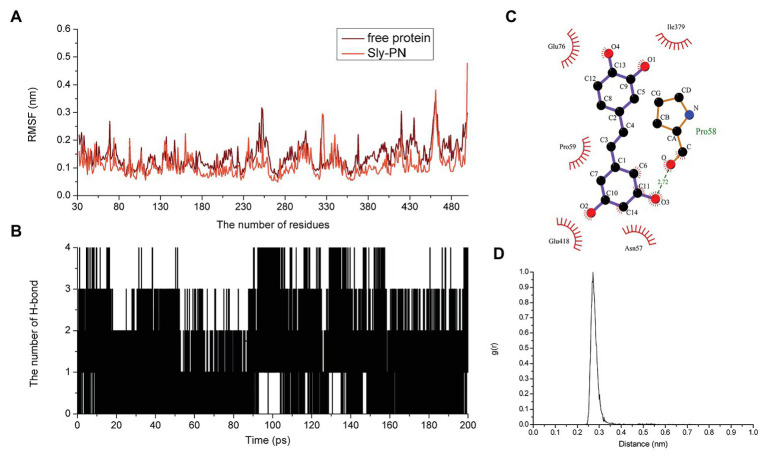
The flexibility of amino acids changed and hydrogen bonds existed in the binding. **(A)** The flexibility of amino acids in the free protein and the PN-SLY complex system. **(B)** The formation of hydrogen bonds during binding. **(C)** The exact details of hydrogen bonds formed between residues in PN and **(D)** the *g*(*r*) value of the acceptor and donor.

Piceatannol bound to the joint region of D2 and D4 in SLY. Both van der Waals forces and electrostatic forces contributed to the interaction between PN and SLY (Figures 3A–C). More concretely, Glu76, Ile379, and Glu380 were close to benzene ring 1 of PN, and strong interactions occurred between them (Figure 3B). Asn57, Pro58, Pro59, and Glu418 were closer to benzene ring 2 of PN (Figure 3B), suggesting that benzene ring 2 was locked into place by Asn57, Pro58, Pro59, and Glu418. Furthermore, the examination of the root-mean-square fluctuations (RMSFs) showed that the residues generated interactions with PN with lower flexibility than those in the free protein, as the RMSF values were less than 0.1 nm (Figure 4A), suggesting that these residues were more rigid (lost flexibility) after binding with PN.

Hydrogen bonding is an essential intermolecular force. Here, we calculated the generation of hydrogen bonds to explore the contribution of hydrogen bonding force to the binding of SLY and PN and found that hydrogen bonding contributed to the binding process between benzene rings 1 and 2, with several fluctuations (Figure 4B). In addition, we confirmed that a hydrogen bond existed between SLY and Main O (Pro58)-O3 in PN by analyzing the details of the interaction with LigPlus software (Figure 4C; Table 2). Furthermore, we calculated the radial distribution function (RDF) values and found that the maximum *g*(*r*) value was observed at 2.00–3.00 Å (Figure 4D), which was consistent with the above results.

**Table 2:** The details of hydrogen bonds between PN and SLY.

Pr.	Acceptor	Donor	Proportion%	Distance(Å)
SLY	Ligand:O3	Pro58Main O	79.71	2.72

### Confirmation of the Binding Sites Between SLY and PN

**Figure 5:**
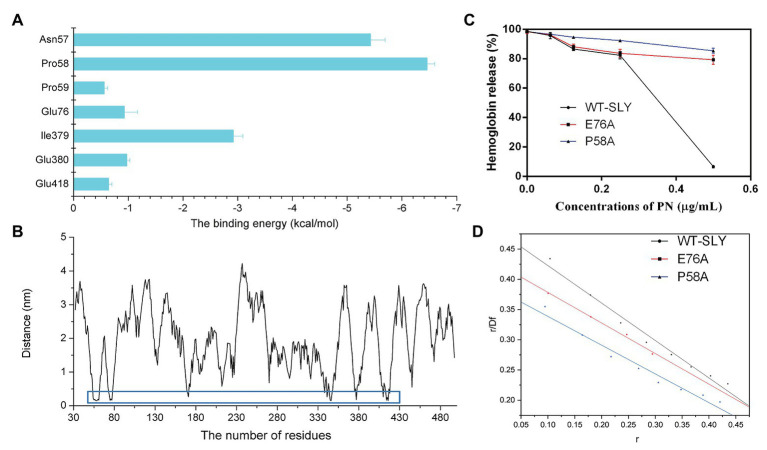
The free energy contribution of residues, and the changes in the pore-forming inhibitory effect and the *K_A_* of PN against SLY mutants. **(A)** The free energy contributions of residues during the binding process of PN and SLY. **(B)** The distance between PN and residues in SLY. **(C)** The inhibitory effect of PN against pore-formation by SLY or its mutant. **(D)** The fitted lines between PN and SLY or its mutants.

To further study the energy contribution of each residue during the binding process, the binding free energy between SLY and PN was calculated by using the MM-PBSA method. Compared with other residues, Asn57 and Pro58 exhibited the most significant binding energy contributions, with *ΔE_total_* values of −5.427 and−6.455 kcal/mol (Figure 5A), respectively, confirming that these two residues contributed greatly to the binding of SLY and PN. In addition, the *ΔE_total_* value of Ile379 was −2.919 kcal/mol (Figure 5A), indicating that Ile379 stabilized benzene ring 1 of PN. Moreover, Glu76 and Glu380 made more substantial binding energy contributions, with *ΔE_total_* values of −0.930 and −0.973 kcal/mol (Figure 5A), respectively, suggesting that Glu76 and Glu380 provided relatively more energy for the interaction between PN to SLY. Furthermore, the *ΔE_total_* values of Pro59 and Glu418 were less than −0.650 kcal/mol (Figure 5A), which indicated that the contributions of Pro59 and Glu418 to the complex system were relatively small. In addition, Pro58 made an essential contribution to the binding of SLY and PN by forming a hydrogen bond between SLY and PN (Figure 4D). Taken together, the results indicate that Asn57, Pro58, Pro59, Glu76, Ile379, Glu380, and Glu418 contributed more energy and were the critical residues during the binding process of PN and SLY. We analyzed the distances between these residues and PN during the last 80 ns of the simulation and found that the distance between the key residues and PN was lower than that for other residues, with values less than 0.3 nm (Figure 5B), verifying the above results.

To verify the key residues involved in the binding of PN with SLY, we performed site-directed mutagenesis on the binding site residues and a MD simulation assay of the P58A and E76A mutants of the PN-SLY complex systems. Simultaneously, a fluorescence spectroscopy quenching (Qiu et al., 2012; Zou et al., 2015) method was carried out to analyze the binding constants (*K_A_*) of the complexes mentioned above, which found that PN almost entirely lost its inhibitory effect against the pore-forming activity of SLY after site-directed mutagenesis of P58A and E76A and the *K_A_* showed consistent trends (Figures 5C,D), providing evidence that Pro58 and Glu76 are central residues during the binding process of PN and SLY.

### Analysis of the Inhibition Mechanism Through Principal Component Analysis

**Figure 6:**
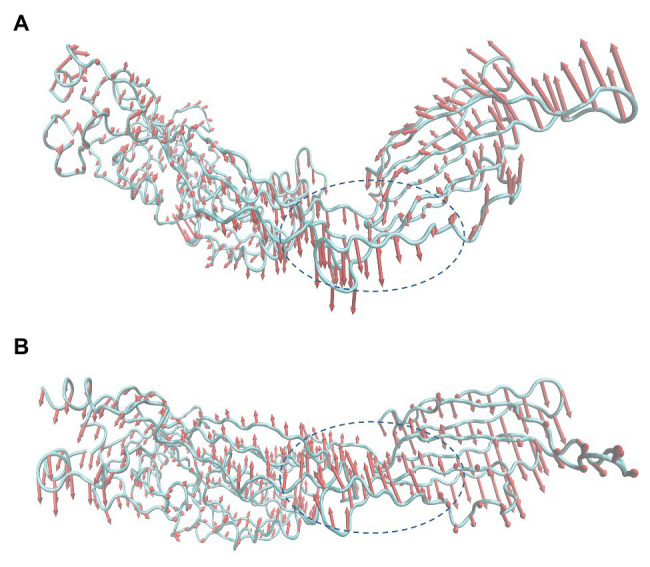
Principal component analysis (PCA) based on the simulation trajectory **(A)** Principal components in free SLY. **(B)** Principal components in the PN-SLY complex. The lengths of the cones represent the magnitude of the motion.

As mentioned before, in the process of forming an oligomer, each monomer must first bind to cholesterol on the host cell membrane. Then, the reaction between monomer and monomer can be activated to form a preformed pore oligomer. In the present study, PN inhibited the hemolytic activity of SLY by restricting the formation of SLY oligomers. Subsequently, principal component analysis (PCA) of free SLY and the SLY-PN complex was performed to explore the key movements of SLY with or without PN. Before binding with PN, there is a specific angle between D2 and D4 of SLY (Figure 6A). After binding with PN, the angle between D2 and D4 of SLY became significantly larger (Figure 6B), indicating that the binding with PN led to changes in the spatial conformation of SLY. According to previous reports, D4 promotes the initial binding of SLY to the cholesterol-containing membrane (Weis and Palmer, 2001; Shimada et al., 2002). Therefore, it is speculated that the binding of SLY and PN resulted in changes in the angle between D2 and D4 of SLY, which led to the inability of SLY to bind cholesterol on the host cell membrane sufficiently. The formation of oligomers was blocked, and eventually, the hemolytic activity of SLY was reduced.

### The Alleviate Effects of PN Against *S. suis* Infections

**Figure 7:**
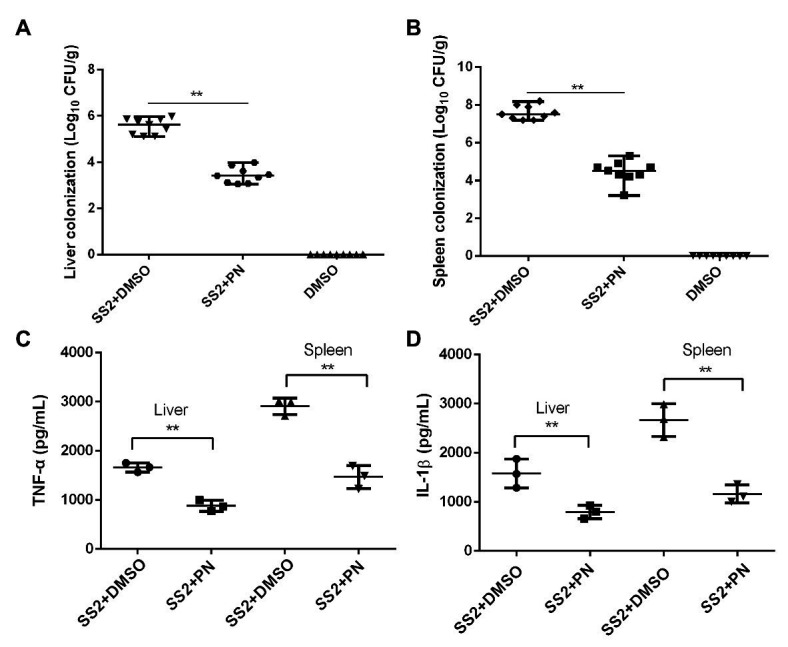
PN protects mice from *S. suis* infection by decreasing inflammation and colony formation. **(A)** *S. suis* colonization in the liver and spleen **(B)**. **(C)** Expression levels of TNF-α and IL-1β **(D)** in the liver and spleen. Data are shown as the mean ± SD, ^**^indicates *p* < 0.01. An unpaired two-tailed *t*-test was used for statistical analysis.

A large number of bacterial colonies were detected in the livers and spleens of mice that were infected with *S. suis*. In contrast, the number of bacterial colonies showed a significant reduction when the infected mice received PN treatment (Figures 7A,B), suggesting that the spread of *S. suis* was partially prevented by PN treatment and resulted in the reduction of colonies. In addition, the inflammatory response was severe in mice infected with *S. suis*, as shown by the detection of a high-level pro-inflammatory factor measured in the liver and spleen. However, this inflammation that was presented with pg/ml (the level of inflammatory factors in the homogenization volume from each individual mouse organ) was alleviated after the infected mice were treated with PN (Figures 7C,D). These results suggested that PN partially alleviated *S. suis* infections in mice by alleviating the inflammatory response and preventing the spread of this pathogen.

## Discussion

Antibiotics are widely used to combat bacterial infection. However, the antibacterial effect is mainly achieved by directly killing bacteria, and the direct consequence is that bacteria develop serious resistance under long-term selective pressure (Balaban et al., 1998; Clatworthy et al., 2011). Virulence factors have been demonstrated to promote pathogen infection and pathogenic ability by enhancing bacteria adhesion and colonization, inducing apoptosis by increasing cytotoxicity and promoting host-pathogen invasion of the host natural barrier (blood-brain barrier or gut barrier; Clatworthy et al., 2011). But many virulence factors are not necessary for bacterial growth. Therefore, anti-virulence strategies are effective alternative measures to combat pathogen infections (Mühlen and Dersch, 2016). SLY is such a toxin in *S. suis*, which makes it an ideal target for the development of inhibitors to combat this pathogen infections.

The cytotoxicity and inflammatory responses caused by SLY have been confirmed, and the cytotoxicity induced by SLY is an important prerequisite for triggering the development of several diseases. Moreover, inflammation occurs simultaneously with *S. suis* infection. Therefore, reducing the cytotoxicity and alleviating or controlling the inflammatory response is an essential aspect to combat *S. suis* infection. Here, we report that PN, as a SLY inhibitor, effectively reduced the cytotoxicity induced by SLY in an *in vitro* coinfection system and alleviated the inflammatory response mediated by *S. suis* both *in vitro* and *in vivo*, providing sufficient evidence that PN could be an ideal lead compound used for combating *S. suis* infection. These results were partly attributed to PN anti-SLY effect but may also be related to the inhibitory effect of PN to spleen tyrosine kinase, which is an important signaling molecule in immune cells. However, the *in vivo* results were only confirmed in mice; in the next work, we will confirm the effect of PN anti- *S. suis* infection on its natural host.

Our team has found that some natural compounds could anti- *S. suis* infection, such as cryptotanshinone (Liu et al., 2020) and formononetin (Wang et al., 2020), but the mechanism was not clear. Our goal is to investigate the relationship between compound structure and the anti-infection effects to determine an optimal structure. Therefore, in this work, we explored the MD based on computational biology and biochemistry methods and confirmed that PN bound to the joint region of D2 and D4 through van der Waals forces and electrostatic forces, and Asn57, Pro58, Pro59, Glu76, Ile379, Glu380, and Glu418 were key residues involved in binding. The spatial conformation of SLY changed after binding with PN, especially the angle change between D2 and D4, causing D4 to not be able to recognize the cholesterol receptor on the cell membrane; science D4 plays a role in identifying receptors and blocking the formation of oligomers; this finally resulted in the loss of hemolytic activity. In our future, we will explore the interaction mechanism of SLY and its other ligands to analyze the relationship between compound structure and the anti-infection effects.

As a food-borne natural compound, PN is widely present in grapes, sugar cane, and other foods, and it has numerous sources, low capital costs, and high safety, making it a promising candidate for combating *S. suis* infections.

## Conclusion

Piceatannol binds to SLY and changes the angle between D2 and D4, which leads to that D4 cannot sufficiently recognize the cholesterol receptor and affects the formation of SLY oligomers, these procedures finally results in the loss of the hemolytic activity. Furthermore, PN reduces SLY-mediated cytotoxicity *in vitro* and alleviates the inflammatory response caused by *S. suis* both *in vivo* and *in vitro*. These results indicate that PN could be used in the future to combat *S. suis* infection.

## Data Availability Statement

The original contributions presented in the study are included in the article, further inquiries can be directed to the corresponding authors.

## Ethics Statement

The animal study was reviewed and approved by Animal Care and Use Committee (ACUC) of Jilin University.

## Author Contributions

HL, TF, and GW designed the study. GW and YG performed the experiments and wrote the paper. XW, XG, and MZ contributed reagents/materials/analysis tools. HL and TF reviewed the manuscript. All authors contributed to the article and approved the submitted version.

### Conflict of Interest

The authors declare that the research was conducted in the absence of any commercial or financial relationships that could be construed as a potential conflict of interest.
